# Supplementary material for: Complex‐centric proteome profiling by SEC‐SWATH‐MS
Source: Mol Syst Biol. 2019 Jan 14;15(1):e8438. doi: 10.15252/msb.20188438 (PMC6346213; doi:10.15252/msb.20188438)
Supplement: Supplementary file 8 — Dataset EV7 [file MSB-15-e8438-s008.zip › feature_plots_string/Q9NVU7.pdf]

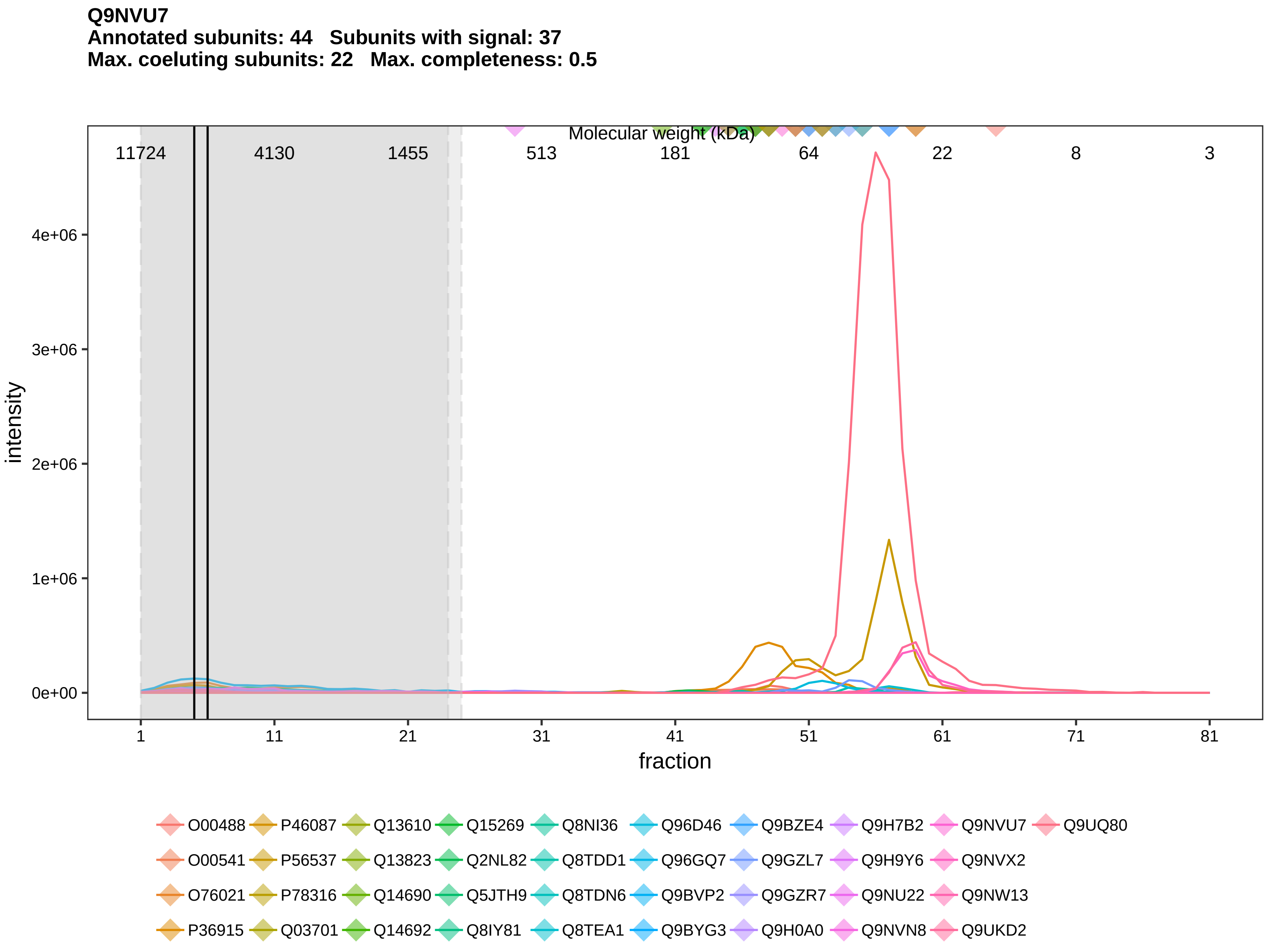
Q9NVU7
Annotated subunits: 44 Subunits with signal: 37
Max. coeluting subunits: 22 Max. completeness: 0.5
Molecular weight (kDa)
11724
4130
1455
513
181
64
22
8
3
intensity
4e+06
3e+06
2e+06
1e+06
0e+00
fraction
1
11
21
31
41
51
61
71
81
O00488
P46087
Q13610
Q15269
Q8NI36
Q96D46
Q9BZE4
Q9H7B2
Q9NVU7
Q9UQ80
O00541
P56537
Q13823
Q2NL82
Q8TDD1
Q96GQ7
Q9GZL7
Q9H9Y6
Q9NVX2
O76021
P78316
Q14690
Q5JTH9
Q8TDN6
Q9BVP2
Q9GZR7
Q9NU22
Q9NW13
P36915
Q03701
Q14692
Q8IY81
Q8TEA1
Q9BYG3
Q9H0A0
Q9NVN8
Q9UKD2
